# Role of Fisetin in Selected Malignant Neoplasms in Women

**DOI:** 10.3390/nu15214686

**Published:** 2023-11-05

**Authors:** Anna Markowska, Michał Antoszczak, Karol Kacprzak, Janina Markowska, Adam Huczyński

**Affiliations:** 1 Department of Perinatology and Women’s Health, Poznań University of Medical Sciences, 60-535 Poznań, Poland; annamarkowska@vp.pl; 2 Department of Medical Chemistry, Faculty of Chemistry, Adam Mickiewicz University, 61-614 Poznań, Poland; michant@amu.edu.pl (M.A.); karol.kacprzak@gmail.com (K.K.); 3 Gynecological Oncology Center, Poznańska 58A, 60-850 Poznań, Poland; jmarkmed@poczta.onet.pl

**Keywords:** fisetin, flavonoids, flavonols, breast cancer, cervical cancer, ovarian cancer, in vitro activity, in vivo activity, nanodelivery systems

## Abstract

A promising therapeutic window and cost-effectiveness are just two of the potential advantages of using naturally derived drugs. Fisetin (3,3′,4′,7-tetrahydroxyflavone) is a natural flavonoid of the flavonol group, commonly found in fruit and vegetables. In recent years, fisetin has gained wide attention across the scientific community because of its broad spectrum of pharmacological properties, including cytotoxic activity against most abundant cancers. By stimulating or inhibiting selected molecular targets or biochemical processes, fisetin could affect the reduction of metastasis or cancer progression, which indicates its chemotherapeutic or chemopreventive role. In this review, we have summarized the results of studies on the anticancer effects of fisetin on selected female malignancies, both in in vitro and in vivo tests, i.e., breast, cervical, and ovarian cancer, published over the past two decades. Until now, no article dedicated exclusively to the action of fisetin on female malignancies has appeared. This review also describes a growing number of nanodelivery systems designed to improve the bioavailability and solubility of this natural compound. The reported low toxicity and activity of fisetin on cancer cells indicate its valuable potential, but large-scale clinical trials are urgently needed to assess real chemotherapeutic efficacy of this flavonoid.

## 1. Introduction

Malignant neoplasms of the genital organs are a significant cause of death among women worldwide, leading to a decrease in average life expectancy [1]. Despite significant advances in both clinical trials and the use of the so-called targeted drugs, the quest for new anticancer agents with high efficacy and low toxicity is continued to prolong and improve the quality of life of female cancer patients [2,3,4]. Oxidative stress, hypoxia, genetic mutations and the lack of apoptotic function are the main internal causes of cancer [5]. Among the promising candidates for new anticancer agents are flavonoids. Both anti-and pro-oxidant properties of flavonoids under normal or pathological conditions, respectively, may be involved in their beneficial effects against cancer, leading to the activation of apoptosis or inhibition of proliferation as well as inflammation [6]. Flavonoid compounds may activate cancer cell death via modulating anti- and proapoptotic proteins or caspases [6]. In addition, both reduction of chronic inflammation and abnormal mitochondrial functions by flavonoids may also be crucial in slowing the progression of cancer [6]. A growing number of studies have indicated that intake of flavonoids in the diet (chemoprevention) can have a beneficial effect on health through the suppression of the development of many diseases, including cancer. It may also contribute to the fight against cancer by lowering the risk of malignancies in various locations, including gynecological cancers [7,8,9,10,11].

**Figure 1:**
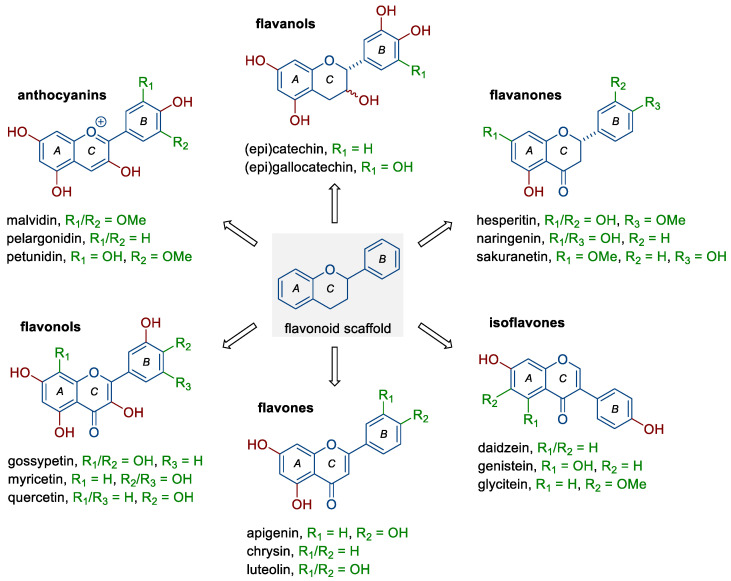
Structure of flavonoids and selected representatives of each of their six subclasses.

Flavonoids belong to a rather large group of plant polyphenols (phytochemicals), which occur naturally in plants and food products of plant origin, including fruit, vegetables, tea, or red wine. They include six subclasses of compounds with distinct chemical structures (Figure 1) and different mechanisms of action, depending mainly on their concentration, route of administration, dose, or type of cancer [6,10,12,13]. The groups mentioned are (i) anthocyanins (e.g., malvidin, pelargonidin, and petunidin), (ii) flavanols (e.g., catechin, epicatechin, and gallocatechin), (iii) flavanones (e.g., hesperetin, naringenin, and sakuranetin), (iv) flavonols (fisetin and, e.g., gossypetin, myricetin, and quercetin), (v) flavones (e.g., apigenin, chrysin, and luteolin), and (vi) isoflavones (e.g., daidzein, genistein, and glycitein). As shown by numerous studies, flavonoids can exhibit pleiotropic anticancer effects; they reduce the proliferation and invasiveness of cancer cells, induce programmed cell death (apoptosis), reduce the activity of pro-inflammatory signaling pathways, as well as inhibit the ability to form new blood vessels (neoangiogenesis) [6,12,14,15,16]. In a meta-analysis based on electronic databases, including a total of five cohort studies and seven case–control studies, Hua et al. [13] noted that dietary flavonoids (flavonols and isoflavones, but not flavones) may have a protective effect against ovarian cancer development (RR 0.82, 95% CI 0.68–0.98). The use of a new type of trans-platinum(II) complex with 3-aminoflavone also resulted in decreased viability and increased mortality of ovarian cancer cells (CAOV3, OVCAR3) [17].

**Figure 2:**
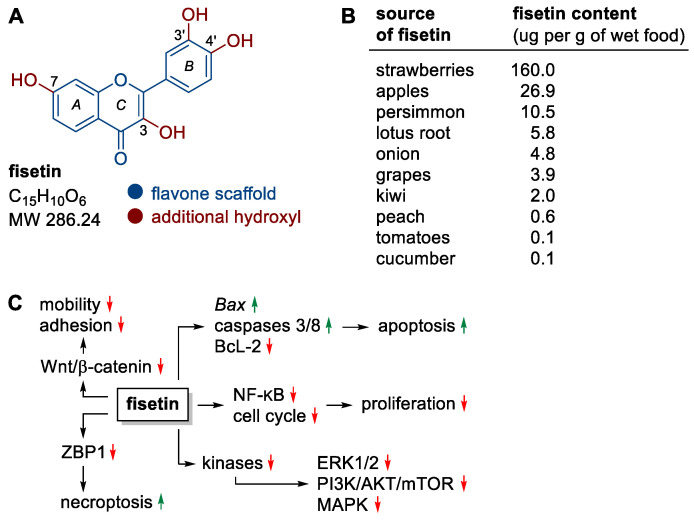
(**A**) Structure of fisetin, (**B**) fisetin content in fruits and vegetables, (**C**) selected molecular targets/processes stimulated (green) or inhibited (red) by fisetin.

Fisetin, one of the representatives of flavonols, deserves special attention in the context of the observed anticancer activity (Figure 2A). The structure of this polyphenol is based on a heterocyclic flavone backbone with additional hydroxyl groups at positions C-3, C-7, C-3′ and C-4′ that are key to the compound’s activity (Figure 2A). The relationship between the structure of fisetin and its activity (SAR) has been studied in detail [18,19]. Particularly important for the flavonoids’ anticancer activity are hydroxyl groups at positions C-5 and C-7 in the A ring or at positions C-3′ and C-4′ in the B ring. Such a substituent pattern may be found in many anticancer active flavonoids [20], including fisetin (Figure 2A) and related compounds. Fisetin that is widely present in medicinal and edible plants has become the focus of many studies, demonstrating its multiple pharmacological effects, such as anti-oxidant, anti-inflammatory, and anticancer in many cell types with no cytotoxic effects on normal cells [21,22]. Thus, fisetin has gained a special attention of researchers as promising chemotherapeutic or chemopreventive agent.

Fisetin is a yellow-ochre dye found naturally in many fruit and vegetables, most notably strawberries, apples, persimmons, onions, grapes and kiwifruit (Figure 2B) [23,24,25]. Currently, this compound is obtained mainly by complex isolation from plant material (about 1 kg of flavonoids mixture is obtained from 0.25 to 0.33 ton of dry fruit or vegetables) [19]. Nevertheless, its chemical synthesis and biotechnological production are being studied intensively [18].

Fisetin can be effective in preventing the development of many types of cancer through its effects on the cell cycle, induction of death, inhibition of (neo)angiogenesis, invasion or metastatic ability of cancer cells, without causing cytotoxic effects on non-cancerous cells [26,27,28,29]. Mechanistically, fisetin affects a wide variety of molecular targets, resulting in a number of anticancer effects, including (i) promoting apoptosis by inducing the expression of the pro-apoptotic genes Bax, caspase 3/8 and down-regulating the expression of the anti-apoptotic protein BcL-2, (ii) promoting necroptosis (a type of programmed cell death morphologically similar to necrosis) by down-regulating ZBP1 protein activity using the threonine–serine kinase pathway RIP1, (iii) inhibiting the activity of the Wnt/β-catenin pathway (e.g., in melanoma and colon cancer cells [30,31]), which is associated with cancer cell motility and adhesion, (iv) inhibition of ERK1/2, PI3K/AKT/mTOR and MAPK signaling pathways through effects on kinases, (v) downregulation of nuclear factor NF-κB and cyclin-dependent cell cycle activity, resulting in the inhibition of cancer cell proliferation (Figure 2C) [26,32,33,34].

The beneficial effects of fisetin against a wide variety of cancers have been unambiguously confirmed, as described by many authors in recent two decades [26,35,36,37,38,39]. However, to the best of our knowledge, no dedicated review covering the research on anticancer activity of fisetin toward cancer cells derived from female malignancies has appeared as yet. Thus, this review focused on fisetin’s anticancer role in breast, cervical, and ovarian cancers.

### Bioavailability of Fisetin

Fisetin, like other flavonoids, has a number of limitations that hamper its widespread use in therapy and supplementation. These include a very low solubility in water (ca. 10 μg mL^–1^), high lipophilicity (log p = 3.2), low chemical stability of its solutions, as well as low bioavailability (44%) with rapid metabolism. For these reasons, many research groups have been working to improve fisetin bioavailability by structure modification or by packing in various nanodelivery systems [40]. Such systems are based on the encapsulation of fisetin in nanoemulsions, liposomes, ethosomes, as well as in polymeric nanoparticles (for reviews, see [41,42,43,44]). More sophisticated solutions use supramolecular aggregates, such as nanocochleates, representing a continuous solid lipid bilayer membrane rolled up in a spiral manner [45] or systems with fisetin encapsulated in lipid micelles introduced into fenugreek galactomannan hydrogel matrix [46]. The latter system is the only one that has so far been tested on humans as a diet supplement. A pharmacokinetic study of a single-dose administered fisetin in both native (1000 mg) and formulated (1000 mg, delivering 192 mg fisetin) forms revealed that the plasma concentration of fisetin over 12 h taken in a delivery system was ~27-fold greater than that when the native form was used [46]. Similarly, the maximum plasma concentration (C_max_) was 23-times higher after administration in a delivery system (238.2 ng mL^–1^) compared to that of the native drug (9.97 ng mL^–1^) [46]. No adverse effects were reported in this study [46]. An interesting approach to improve the performance of anticancer lobaplatin was its co-crystallization with a variety of flavonoids, including fisetin, luteolin, myricetin, naringenin, and quercetin [47]. The obtained cocrystals showed reduced solubilities, slower-release rates, and delayed hydrolysis compared to those of lobaplatin, but the solubilities and dissolution rates of the flavonoids were significantly improved [47]. CCK-8 assay, used for the evaluation of anticancer activity, has shown that the cocrystals of lobaplatin-fisetin, -luteolin, and -myricetin exhibited superior performances to lobaplatin itself [47].

For more information on fisetin delivery nanosystems and their activity in broad cancer chemotherapy, see a recent review article [38]. Here, we discuss promising formulations in the context of their application against female malignancies. The findings collected in this review, as part of a series of papers describing the effects of nutrients on the course of female malignancies [48,49,50,51], should be interesting for a broad audience, especially researchers, physicians, and patients struggling with the types of cancer in question. The presented material has been extracted from Google Scholar, PubMed, and Scopus databases, and covers original papers on the anticancer activity of fisetin in in vitro/in vivo assays (Table 1), as well as its nanodelivery systems (Table 2) that have been published in the last 20 years.

## 2. Breast Cancer

Breast cancer (BC) is the most frequently diagnosed malignancy in women worldwide. According to the GLOBOCAN global registry, the cancer ranks first in both incidence and mortality (24.5% and 15.5%, respectively) [1]. BC needs, besides prevention, an intense research on the development of new therapies as well as constant expansion of the arsenal of novel oncological drugs [54]. The reports on the potential effects of fisetin have mostly concerned BC (Table 1), much more often than other malignant neoplasms. Also, the design, construction and use of fisetin delivery nanosystems have been mostly BC-directed (Table 2). In BC, epigenetic regulation of human epidermal growth factor receptor 2 (HER-2) is observed, and in this context, fisetin was effective on BC cell lines overexpressing HER-2/neu receptor [35]. On the other hand, the PI3K/AKT/mTOR signaling pathway is activated in ~70% of BCs, and correlated with clinical characteristics and poor prognosis [77,78]. Fisetin has been found to target as well as inhibit this signaling pathway [54].

### In Vitro and In Vivo Activity of Fisetin

Some BCs show overexpression of the HER-2 receptor, which sometimes results in the development of cancer cells’ resistance to the chemotherapeutic drugs used (HER-2 inhibitors). In this context, an in vitro study by Guo et al. [52] has demonstrated on MDA-MB-453 BC cells with HER-2 receptor overexpression that fisetin is capable of inducing apoptosis, inactivating the HER-2 receptor, degrading the proteasome or altering phosphorylation of the PI3K/AKT signaling pathway. Similarly beneficial effects of fisetin on HER-2^+^ SK-BR-3 cells have also been confirmed [53].

Another study has concerned the activity of fisetin on BC cell lines (4T1, MCF-7, MDA-MB-231) and in vivo tests on BC heterotransplantation (4T1) in mice [54]. It has been shown that fisetin can reduce the proliferation and invasiveness of cancer cells, as well as their potential to metastasize and, in an animal model, inhibit the growth of cancerous tumors [54]. Molecular studies indicated that these effects depended on the PI3K/AKT/mTOR pathway, which affects cancer malignancy [54]. In a comparative study, fisetin was more cytotoxic against MCF-7 cells than MDA-MB-231, while having no significant effects against the non-cancerous MCF-10A cells used in the assays [55].

Metastasis is commonly observed in advanced BC and is one of the leading causes of death among patients with malignancies. In BC with such characteristics, extracellular matrix metalloproteinases (MMPs), critical regulators of the metastatic process, are often overexpressed, and fisetin can affect the downregulation of MMP-2/9 enzymes by the multifunctional nuclear regulator Nrf2 and heme oxygenase 1 (HO-1) [56]. Fisetin’s ability to reduce the invasiveness of MCF-7 cancer cells may also result from the inhibition of MMP-9 enzyme activation via the PKC/ROS/MAPK pathway [57].

About 10–15% of BCs are triple-negative BCs (TNBCs) [79], characterized by an aggressive outcome course. Unfortunately, despite the introduction of new immune treatments, they mean a poor prognosis for patients. Using two TNBC lines (BT549, MDA-MB-231), Li et al. [59] have shown that fisetin, in dose depended fashion, can inhibit the proliferation, migration, and metastasis of these cells. In contrast, the use of fisetin in in vivo studies resulted in inhibition of primary tumor growth, but also reduced lung metastasis [59]. The reduced ability of metastatic TNBC cells to migrate may be due to interference with the activity of oncogenic protein kinases [60]. Fisetin activity was also mediated by the PTEN/AKT/GSK-3β pathway, which can reverse the epithelial–mesenchymal transition (EMT) process [59]. In another study by Smith et al. [53], fisetin inhibited not only the growth of MDA-MB-231 and MDA-MB-468 cells, but also their ability to form colonies without much effect on the growth of non-cancerous cells. Worth noting is that the cytotoxic effect of 5-fluorouracil, cisplatin and the 4-hydroxycyclophosphamide metabolite of cyclophosphamide on TNBC cells was enhanced in the presence of this flavonol [53]. A significant synergistic effect (*p* < 0.01, CI < 1.0) between fisetin and another flavonoid, quercetin, was observed for a variety of BC cell lines (4T1, BT549, MCF-7, MDA-MB-231, and T47D) and in an animal model, leading in this case to inhibition of tumor progression [61].

With regard to TNBC, in addition to surgery and/or chemotherapy, radiation remains an essential strategy for fighting the cancer. Radiation therapy causes DNA damage, unfortunately, very often loses its effectiveness due to radioresistance, one reason for which is the upregulation of Y-box binding protein 1 (YBX1) activity. Similarly to ionizing radiation, fisetin caused double-strand breaks (DSBs) and chromosomal aberrations of cancer cells [62]. Interestingly, the use of fisetin interfered with the repair of DSBs induced by irradiation directed cancer cells toward their programmed death [62]. This may indicate that the use of this flavonol contributes to improvement of the outcome of TNBC in patients treated with radiotherapy.

The use of nanotechnology can improve the bioavailability of fisetin, which may, in turn, increase its clinical efficacy. Fisetin was embedded in poly(lactic acid) (PLA) nanoparticles to increase its solubility and activity [70]. Cytotoxicity tests conducted on BC heterotransplantation (4T1) in mice have proved that the anticancer activity of such nanoparticles is superior to that exhibited by free fisetin [70]. On the other hand, in another study, encapsulation of fisetin complex with hydroxypropyl-β-cyclodextrin (HPβCD) into poly(lactide-co-glycolic acid) (PLGA) nanoparticles not only improved the bioavailability of the flavonol after oral administration (as evidenced by increased maximum plasma concentration and total drug absorption), but also increased the anticancer activity and ability to induce apoptosis in MCF-7 cells [71]. The anticancer effects of fisetin in in vitro and in vivo tests have also been described by Wang et al. [72], who compared the effects of the activity of free fisetin and its polymeric α-tocopherol-based micelles. Free fisetin induced apoptosis of BC cells in about 11%, whereas, in micellar form, this percentage increased to nearly 20% [72]. After 48 h, apoptosis with free fisetin increased to 30%, while that with vehicle-packed fisetin reached 42% [72]. The authors of this study also observed a significant reduction in tumor size in in vivo studies [72]. Beneficial effects, such as increased bioavailability and anticancer activity, have also been noted for fisetin-loaded dimyristoyl phosphatidylcholine liposomal vesicles converted into nanocochleates by the action of Ca^2+^ ions [45], fisetin-loaded folate functionalized pluronic micelles [73] or fisetin encapsulated into lipoprotein bioinspired lipid nanoparticles (LPINs) [74]. On the other hand, human serum albumin (HSA) nanoparticles could also be used as carriers for various flavonoids, including fisetin, to deliver them to specific locations, to contribute to selectively affect cancer cells [75].

## 3. Cervical Cancer

Cervical cancer (CC) is among the most frequently diagnosed malignancies in women. It ranks fourth in incidence, after breast, colorectal, and lung cancer, and accounts for 6.5% of all malignancies [1]. Therefore, it is extremely important to develop new strategies to combat this type of cancer. Many reports point to a therapeutic role of diverse flavonoids, including fisetin, in the fight against CC, as described later in this chapter. The arachidonic acid signaling pathway plays one of the key roles in this type carcinogenesis. At the same time, flavonoids, by inhibiting this pathway, could be used in chemoprevention and/or anticancer therapy [80]. The effects of fisetin on CC only have been observed in in vitro and in vivo studies (Table 1). Reports regarding the use of nanotechnology to increase the bioavailability and anticancer activity of fisetin have been very limited so far (Table 2). Among the various components of the plasminogen system, urokinase-type plasminogen activator (uPA), together with its receptor, play a pivotal role in cancer progression and metastasis, while uPA is regulated through the MAPK or PI3K-AKT signaling pathways in CC [26]. Importantly, fisetin has been found to downregulate the expression of the uPA gene through the selective inhibition of the p38 MAPK-dependent NF-κB signaling pathway [58].

### In Vitro and In Vivo Activity of Fisetin

Fisetin activates CC cell death, which was confirmed by the reduced growth of these cells, changes in their morphology, or cell cycle arrest in the G2/M phase [63]. Chou et al. [64] have quantified the effect of fisetin on invasion and migration of CC cells. Addition of this compound to the cell culture, reduced motility of SiHa and CaSKi cells by 46.0% and 81.0%, respectively, when used at a concentration of 20 µM, while at a doubled concentration, the analogous reduction was 62.1% and 90.2%, respectively, in line with reduction of cell invasiveness (84.2% for SiHa and 92.4% for CaSKi) [64]. Mechanistically, the anticancer activity of fisetin was associated with the inhibition of p38 MAPK phosphorylation and interference with NF-κB factor translocation, resulting in the decreased expression of uPA, which affected extracellular matrix degradation, facilitating the invasion or increasing the potential for metastasis [64]. Additionally, Afroze et al. [65] have determined the effect of fisetin on the mechanisms of proliferation as well as programmed death in HeLa cells. Application of fisetin modulated the activity of a number of proapoptotic (APAF1, Bad, and Bax) and anti-apoptotic (BcL-2, BIRC, MCL-1, and XIAP) genes, as well as arrested the cell cycle in the G2/M phase [65] (similarly to [63]). As a result, the expression of AKT/MAPK pathways was downregulated, leading to apoptosis of cancer cells [65]. In addition, a reduction in inflammation was observed through regulation of the JAK-STAT/NF-κB pathway [65]. In another study, using the HeLa cell line, fisetin affected cell proliferation and apoptosis in a dose- and time-dependent manner [58]. Fisetin acted here as an activating factor for caspase 3/8 and ERK1/2 phosphorylation [58].

In a different approach, Lin et al. [66] used fisetin in combination with the multikinase inhibitor sorafenib. In their in vitro and in vivo studies, synergistic effect on enhancing apoptosis, mainly by affecting caspase 3/8 or increasing Bax expression has been observed [66].

Literature reports on the use of nanodelivery systems to enhance the activity of fisetin against CC cells, are rare and essentially limited to a study by Xiao et al. [12]. The use of fisetin, as well as fisetin micelles at a concentration of 30 µM, resulted in a decrease in CaSki cell viability; cell survival curves after using fisetin and fisetin micelles for up to four days showed >50% inhibition ratio compared to control groups [12]. However, the authors of the study did not compare the activity of the free fisetin and fisetin-loaded nanodelivery systems.

## 4. Ovarian Cancer

Ovarian cancer (OC) has the worst prognosis of all gynecological cancers. In most cases, this type of cancer is diagnosed in advanced clinical stages; nearly 80% of patients experience recurrences, significantly reducing the five-year survival rate of 25–30% [1,81]. In light of these very unfavorable statistics, it is an extremely timely task for researchers and physicians to search for new therapies that can help improve the quality, as well as prolong the life of patients struggling with this type of cancer. The use of fisetin may be an interesting option in this context, both due to its activity against OC in vitro and in vivo (Table 1) as well as the use of nanodelivery systems to improve the anticancer potential of the native flavonoid drug (Table 2).

### In Vitro and In Vivo Activity of Fisetin

One of the reasons for the occurrence of relapses, is the development of chemoresistance in cancer cells, which is mediated by a specific tumor microenvironment containing a pool of self-renewing cancer stem cells (CSCs). Resistance to cytostatics also results from the lack of activity of the ERK signaling pathway, which mediates mitochondrial cytochrome c release or cell cycle arrest, among other processes. In this context, Koren Carmi et al. [32] have demonstrated in vitro that OC cells cultured together with human stem cells are not susceptible to chemotherapeutic agents as a result of blocking the ERK pathway. Importantly, fisetin actively restored ERK phosphorylation, thereby sensitizing the cells to the applied treatment [32].

Abd Ghani et al. [28] have conducted a study on the affinity and interaction between flavonoids, including fisetin, and anti-apoptotic proteins (BcL-2, BcL-XL). The molecular method used (molecular docking) has shown that fisetin, acting as an inhibitor of these proteins, can induce programmed cancer cell death [28]. Western blot analysis by Meng et al. [68] has confirmed that fisetin exhibits antiproliferative properties against SKOV3 cells. Higher concentrations led, however, to a significant decrease in BcL-2 and an increase in Bax [68]. In an animal model, tumor volume and tumor mass were also significantly reduced [68]. According to the authors of another study, fisetin, depending on the dose used (the optimal concentration was 100 µM), can lead not only to apoptosis but also necroptosis of cancer cells (A2780, and OVCAR3) [34]. A key molecular target for necroptosis was the DNA-binding protein ZBP1 [34]. The use of fisetin nanoparticle proved to be an interesting strategy for improving the activity of fisetin against OC cells, leading to lower IC_50_ parameter values (125–250 μg mL^–1^ for fisetin and 62.5–125 μg mL^–1^ for fisetin nanoparticles, respectively) [69].

On the other hand, an increasing number of literature reports indicate a beneficial anticancer effect of fisetin in combination with common oncology drugs, related to reduction of the chemoresistance of OC cells. Fisetin, administered in combination with cisplatin, induced apoptosis of A2780 cells [33]. In an animal model, inhibition of signaling, including the mTOR pathway, and induction of cancer cell death by increasing expression of the proapoptotic gene Bax and activation of caspase 3/9 have been reported [33].

A nanosized delivery system for an improved bioavailability of fisetin against OC cells was first implemented by Xiao et al. in 2018 [12]. The use of polymeric micelles (10~100 nm) reduced the release rate of fisetin (73% versus 93%), but also more effectively induced apoptosis in in vivo tests on OC heterotransplantation (SKOV3) in mice compared to those of free fisetin [12]. The nanodelivery approach has also proved beneficial for fisetin in combination with paclitaxel against OC cells (CAOV3, OVCAR3) [76]. Both components were placed inside nanoparticles formed by a naturally occurring polysaccharide (starch), coated with folate-conjugated poly(ε-caprolactone)/poly(ethylene glycol) copolymer [76]. Such nanoparticles show an affinity to vitamin (folate) receptors rapidly accumulating inside OC cells and induce their death at much lower doses than with fisetin and paclitaxel used separately [76]. From a mechanistic point of view, the use of nanoparticles induced an increase in Bax and Bid, caspase 3, poly(ADP-ribose) polymerase (PARP) genes, as well as a decrease in anti-apoptotic genes (BcL-XL, MCL-1) [76]. The use of nanotechnology to improve the therapeutic potential of fisetin has also been confirmed by other authors [16].

## 5. Conclusions

Despite significant advances in its diagnosis and therapy, cancer is the leading cause of death in developing countries. Therefore, the search for new anticancer drug candidates is of fundamental importance. Naturally derived compounds constitute a large pool of anticancer candidates that offers many opportunities, but also challenges. Fisetin is a bioactive polyphenol belonging to flavonoids, which can affect a wide variety of signaling pathways related to cell division, inflammation, or oxidative stress, among others. Disease prevention is always better than disease treatment, in this context literature reports treating the multidirectional activity of fisetin, for example, on myoma cells—the most common benign uterine cancer—are of interest in chemoprevention.

In this review, we have compiled the results of in vitro and in vivo studies on the effect of fisetin against selected malignancies in women, i.e., breast, cervical, and ovarian cancers. Of note is that the number of published reports describing the activity of fisetin varies significantly depending on different types of cancer. While studies on breast cancer cells in in vitro and in vivo tests have been comprehensively described by various research teams, the number of articles reporting results on cervical or ovarian cancer cells is significantly smaller. Further studies should be concerned with the detailed effects of fisetin on different cancer (sub)types. In addition, a deeper understanding of the impact of fisetin on the tumor microenvironment or its immunomodulatory properties seems to be important for therapy.

**Figure 3:**
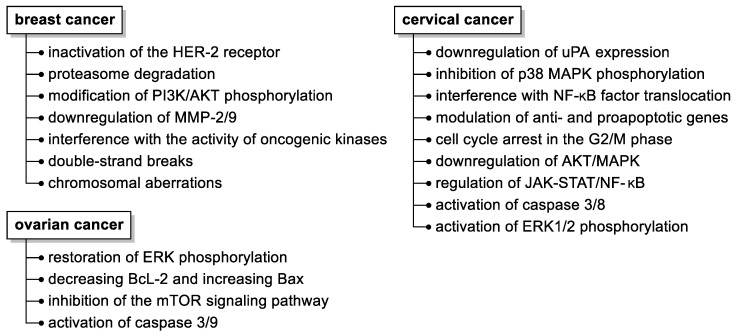
Summary of the anticancer activities of fisetin in selected malignant neoplasms in women. Abbreviations used: HER-2, human epidermal growth factor receptor 2; MMP, matrix metalloproteinase; uPA, urokinase-type plasminogen activator.

Although several molecular mechanisms of fisetin action have been elucidated (Figure 3), such as its pleiotropic effects on a variety of molecular targets, inhibition of cancer development by affecting cell cycles, programmed death, and hampering (neo)angiogenesis, invasion, or metastatic capacity, the precise molecular targets and signaling pathways affected by fisetin still need to be disclosed.

Only single reports can be found on other gynecological cancers, such as endometrial cancer, therefore it seems reasonable to add to the body of knowledge in this area. Understanding the pharmacokinetics and toxicity profiles of fisetin seems also to be essential for its clinical application.

In contrast to preliminary medical studies on fisetin, much better results have been reported on its formulation and nanodelivery systems. These studies applied to fisetin and other flavonoids roughly solved the problem of their low solubility, stability and bioavailability, thus opened a wider perspective for reliable research and clinical trials. Future studies in this direction should also focus on standardization of fisetin form and delivery system that would ensure high bioavailability and stability as well as determine optimal therapeutic concentrations regarding particular cancer treatment.

The potential use of fisetin in chemopreventive management, adjuvant treatment, or synergy in combination with common cancer drugs also requires further verification, including long-term effects (therapeutic versus adverse) and optimal dosage regimens. Further exploration of such combination strategies, including those with metabolic inhibitors, may help optimize treatment options or overcome potential mechanisms of drug resistance. Two clinical trials are currently planned (NCT04733534 and NCT05595499, clinicaltrials.gov), which would aim to assess the efficacy of using fisetin to improve the physical performance of people with an oncological history, including postmenopausal women who have received chemotherapy for stage I–III breast cancer. In the light of the exploratory studies described in this review, it seems that we are already well prepared to reliably use fisetin and other flavonoids to fight civilization-related types of cancer.

## Figures and Tables

**Table 1:** In vitro (and animal) studies with fisetin on cancer cell lines.

Cancer Type	Cancer Cell Lines	Fisetin Concentration (µM)	Combination Treatment and/or In Vivo Experiments	Ref.
Breast cancer	MDA-MB-453	20, 40, 60, 80, 100		[52]
	MCF-7, SK-BR-3, MDA-MB-231/468	25, 50, 100	4-hydroxycyclophosphamide, 5-fluorouracil, cisplatin	[53]
	4T1, MCF-7, MDA-MB-231	20, 40, 80	animal tests (223 mg kg^–1^ fisetin administered intraperitoneally)	[54]
	MCF-7, MDA-MB-231	25, 50, 100, 200		[55]
	4T1, JC	1, 3, 5, 10		[56]
	MCF-7	1, 5, 10, 20, 30, 50		[57]
	MDA-MB-231	10, 20, 40, 60, 80		[58]
	BT-549, MDA-MB-231	10, 30, 100	animal tests (100 mg kg^–1^ fisetin administered intraperitoneally)	[59]
	BT-20/549, Hs578T, HCC70/1806/1937, MDA-MB-157/231/468	0–200	animal tests on zebrafish (6.25–100 µM fisetin)	[60]
	4T1, BT-549, MCF-7, MDA-MB-231, T47D	10–250	quercetin, animal tests (220 mg kg^–1^ fisetin + 200 mg kg^–1^ quercetin; the dosage was doubled for groups that received individual treatment)	[61]
	Hs578T, MCF-7, T47D, MDA-MB-231/453/468	12.5, 25, 50, 75	irradiation (1 Gym min^–1^ dose rate)	[62]
	MCF-7	5, 10, 25, 50, 100, 250		[63]
Cervical cancer	CaSki	10, 30, 100, 300		[12]
	HeLa	10, 20, 40, 60, 80	animal tests (2/4 mg kg^–1^ fisetin administered intraperitoneally)	[58]
	HeLa	5, 10, 25, 50, 100, 250		[63]
	CaSki, SiHa	10, 20, 40		[64]
	HeLa	1, 10, 20, 30, 35, 40, 45, 50, 55, 60, 65, 70		[65]
	HeLa	5, 10, 20, 40, 80	sorafenib, animal tests (4 mg kg^–1^ fisetin alone administered orally or 4 mg kg^–1^ fisetin + 10 mg kg^–1^ sorafenib administered orally)	[66]
Ovarian cancer	A2780, A2780CisR	10	platinum(IV) prodrug RJY13 (please refer to [67])	[32]
	A2780	50, 75, 100, 125, 150, 200 ^1^	cisplatin	[33]
	A2780, OVCAR3	25, 50, 100		[34]
	SKOV3	25, 50, 100, 200, 400	animal tests (200/400 mg kg^–1^ fisetin)	[68]
	SKOV3	3.93, 7.81, 15.63, 31.25, 62.5, 125, 250, 500, 1000 ^1^	animal tests (1.25 mg kg^–1^ fisetin or fisetin nanoparticle)	[69]

Fisetin concentration at µg mL^–1^.

**Table 2:** In vitro (and animal) studies with fisetin-loaded nanodelivery systems on cancer cell lines.

Cancer Type	Cancer Cell Lines	Nanodelivery System	Advantage over Free Fisetin	In Vivo Experiments	Ref.
Breast cancer	MCF-7	DMPC liposomal vesicles converted into nanocochleates	increased anticancer activity, higher fisetin plasma concentrations		[45]
	4T1 (only in vivo)	PLA NPs	increased antitumor effect, reduced tumor volume, lower toxicity	40 mg kg^–1^ fisetin-loaded PLA NPs administered intravenously	[70]
	MCF-7	HPβCD complexes incorporated into PLGA NPs	higher cytotoxicity and increased cellular uptake efficiency		[71]
	MCF-7	TPGS-PLA polymeric micelles	higher internalization, enhanced cytotoxicity, reduced tumor volume	no information on both concentrations and route of administration	[72]
	MCF-7	folate functionalized pluronic micelles	improved anticancer activity, higher fisetin plasma concentrations, reduced systemic toxicity		[73]
	MDA-MB-231	lipoprotein-inspired NPs and their biopolymer coated nanosystems	increased cytotoxicity, superior inhibition of tumor growth	equivalent fisetin concentration of 10 mg kg^–1^ administered intravenously	[74]
	MCF-7	human serum albumin NPs	selective toxicity to cancer cells, increased solubility and stability		[75]
Cervical cancer	CaSki	polymeric micelles	not compared (both fisetin and fisetin micelles decreased cancer cell viabilities)		[12]
Ovarian cancer	SKOV3	polymeric micelles	increased in vitro antiproliferative activity, marginally stronger antitumor activity	100 mg kg^–1^ of fisetin micelles administered intraperitoneally	[12]
	SKOV3	flavonoid NPs	not compared (the use of fisetin NPs resulted in decreasing cell proliferation and increasing apoptosis)	1.25 mg kg^–1^ of fisetin NPs administered intravenously	[16]
	CAOV3, OVCAR3	folate conjugated PCL-PEG copolymer coating of the polysaccharide NPs	induction of cancer cell death at lower doses, more rapid internalization		[76]

DMPC, dimyristoylphosphatidylcholine; HPβCD, hydroxypropyl-β-cyclodextrin; NPs, nanoparticles; PCL-PEG, poly(ε-caprolactone)/poly(ethylene glycol); PLA, polylactide; PLGA, poly(lactide-co-glycolide); TPGS, D-α-tocopheryl polyethylene glycol succinate.

## Data Availability

Not applicable.
